# Sphingosine 1-Phosphate Regulates Obesity and Glucose Homeostasis

**DOI:** 10.3390/ijms25020932

**Published:** 2024-01-11

**Authors:** Kazuo Kajita, Isao Ishii, Ichiro Mori, Motochika Asano, Masayuki Fuwa, Hiroyuki Morita

**Affiliations:** 1 Department of Health and Nutrition, Faculty of Home Economics, Gifu Women’s University, 80 Taromaru, Gifu 501-2592, Japan; 2 Department of Health Chemistry, Showa Pharmaceutical University, 3-3165 Higashitamagawagakuen, Machida 194-8543, Japan; 3 Department of General Medicine and General Internal Medicine, Graduate School of Medicine, Gifu University, 1-1 Yanagido, Gifu 501-1194, Japan; m980897@gmail.com (I.M.); motoasa0201@yahoo.co.jp (M.A.); f_m_v_v@yahoo.co.jp (M.F.); morita.hiroyuki.d6@f.gifu-u.ac.jp (H.M.)

**Keywords:** adipocyte, adipogenesis, ceramide, glucose tolerance, inflammation, insulin resistance, lipotoxicity, S1P receptor, sphingosine kinase, sphingosine 1-phospahte

## Abstract

One of the major global health and welfare issues is the treatment of obesity and associated metabolic disorders, such as type 2 diabetes mellitus and nonalcoholic fatty liver disease. Obesity, caused by the excessive accumulation of triglycerides in adipose tissues, induces adipocyte dysfunction, followed by inflammation, in adipose tissues and lipotoxicity in nonadipose tissues. Several studies have shown that obesity and glucose homeostasis are influenced by sphingolipid mediators, including ceramide and sphingosine 1-phosphate (S1P). Cellular accumulation of ceramide impairs pancreatic β-cell survival, confers insulin resistance in the liver and the skeletal muscle, and deteriorates adipose tissue inflammation via unknown molecular mechanisms. The roles of S1P are more complicated, because there are five cell-surface S1P receptors (S1PRs: S1P_1–5_) which have altered functions, different cellular expression patterns, and inapparent intracellular targets. Recent findings, including those by our group, support the notable concept that the pharmacological activation of S1P_1_ or S1P_3_ improves obesity and associated metabolic disorders, whereas that of S1P_2_ has the opposite effect. In addition, the regulation of S1P production by sphingosine kinase (SphK) is an essential factor affecting glucose homeostasis. This review summarizes the current knowledge on SphK/S1P/S1PR signaling in and against obesity, insulin resistance, and associated disorders.

## 1. Introduction

Obesity is one of the most common medical and welfare problems worldwide. According to the World Health Organization (WHO), >1.9 billion adults worldwide were overweight (body mass index [BMI]: ≥25) in 2016, including 650 million who were obese (BMI: ≥30), with the prevalence of obesity tripling between 1975 and 2016. Obesity is associated with metabolic syndrome, type 2 diabetes mellitus (T2DM), hypertension, dyslipidemia, hepatic steatosis, nonalcoholic fatty liver disease (NAFLD), chronic kidney disease, and a few forms of cancer. Many metabolic diseases are associated with insulin resistance. Lifestyle improvement is important for treating obesity, and considerable efforts have been made to highlight the importance of diet and exercise. However, transforming all patients into healthier conditions is difficult, especially in modern societies with the prevalent overnutrition and lack of exercise that now occurs in developed and developing countries. Therefore, there is a compelling need for alternative strategies to treat obesity, such as easy-to-administer oral therapeutics. Among the numerous candidates, a few researchers have focused on sphingolipid-derived mediators such as sphingosine 1-phosphate (S1P) and ceramide, which have potent and diverse physiological properties. In this review, we summarize the biological roles of S1P/ceramide in obesity and insulin signaling and discuss the prospects of S1P receptor (S1PR) agonists for use as therapeutics, partly based on our results [1,2], where obesity and T2DM could be treated by modulating specific S1PR signaling. Additionally, there is accumulating evidence showing the roles of S1P/S1PR in the onset and progression of NAFLD and hepatic fibrosis [3,4,5]. However, this has not been discussed herein because of the current lack of clear relevance to human pathology compared to obesity. We have also deferred a discussion on the roles of S1P/S1PR in cardiovascular, kidney, and inflammatory diseases that has been described in excellent reviews [6,7,8].

## 2. Adipocyte and Obesity

### 2.1. Adipocyte Proliferation and Differentiation

Obesity develops when excess energy is stored in adipocytes as triglycerides (neutral fats). Adipocytes are derived from mesenchymal stem cells and specialize in triglyceride storage. Their cell bodies appear to be occupied by lipid droplets. As the largest endocrine organ in the body, the adipose tissue secretes hormones that regulate glucose, lipid metabolism, and cytokines that regulate systemic inflammation.

Excessive nutrition can increase the size (hypertrophy) and number (hyperplasia) of adipocytes in the adipose tissue. Much evidence supports the idea that adipocyte hypertrophy leads to the onset of insulin resistance and metabolic disorders [9,10]. Adipocytes are classified based on their anatomical location, with the most representative examples being visceral adipocytes, including epididymal, intestinal, and perirenal adipocytes, and subcutaneous adipocytes, such as inguinal adipocytes, although adipocytes exist in the skin, skeletal muscle, pericardium, breast, and bone marrow. Obese visceral adipose tissue (VAT) is considered harmful, whereas obese subcutaneous adipose tissue (SAT) is not [11]. Even when body weight increases similarly, metabolic disorders largely differ depending on whether this increase is associated with adipocyte hypertrophy or hyperplasia or is mainly caused by obesity in the VAT or SAT. The process of how they are determined is mostly unknown; however, understanding “adipogenesis” provides certain clues.

Adipogenesis comprises two phases: determination and terminal differentiation. In the determination phase, mesenchymal stem cells enter the adipocyte lineage through a stimulus called “adipogenic commitment” to form preadipocytes [12]. Although much about this process remains unknown, the route to preadipocytes has been examined by cell lineage tracing. Delta-like homolog 1 (DLK1), a non-canonical Notch ligand, was the first identified marker for preadipocytes [13]. After that, several cell surface preadipocyte markers were identified. Two cell populations, CD24^+^ (Lin^−^, CD34^+^, CD29^+^, Sca-1^+^, and CD24^+^) and CD24^−^ (Lin^−^, CD34^+^, CD29^+^, Sca-1^+^, and CD24^−^), were selected from platelet-derived growth factor receptor α (PDGFRα)-positive stromal vascular cells (SVC) in white adipose tissue [14,15]; however, these markers have been detected in both stem cells and hematopoietic cells, and more specific markers are anticipated. For these situations, we proposed “proliferin” as a novel marker of small proliferative adipocytes, namely, possible beige cell progenitors (preadipocytes) [16]. In the second terminal differentiation phase, fibroblast-like preadipocytes transform into round, lipid-laden cells by expressing several adipocyte-specific genes [17].

Visceral and subcutaneous adipocytes were initially believed to be identical and to differ only in their anatomical locations and systemic effects [18]. However, the Wilms tumor gene, WT1, is specifically expressed in visceral preadipocytes [19]. A study using AdipoChaser mice fed high-fat diets (HFDs) revealed that epididymal adipocytes initiated adipogenesis by 4 weeks. In contrast, subcutaneous adipocytes underwent hypertrophy only after 12 weeks [20]. In addition, visceral adipocytes emerged postnatally, whereas subcutaneous adipocytes developed mainly between embryonic days 14 and 18 [21]. Thus, visceral adipocytes are substantially different from subcutaneous adipocytes, and single-cell sequencing has recently been used to reveal heterogeneity in adipogenic cells [21,22]. Currently, no ideal preadipocyte marker is common to all adipocytes but not expressed in other progenitor cells [23], and the determinant(s) of adipocyte hypertrophy or hyperplasia remain uncharacterized. Treatment with thiazolidine converts adipocytes from hypertrophy to hyperplasia [10]. Genetic deletion of the transmembrane BAX inhibitor motif containing 1 (TMBIM1), an inhibitor of adipogenesis, induces visceral adipocyte hyperplasia and improves obesity-related metabolic diseases in HFD-fed mice [24]. The distribution of excess nutrients between the visceral and subcutaneous adipocytes is not well understood. However, it is influenced by sex hormones [25].

### 2.2. Lipotoxicity

**Figure 1:**
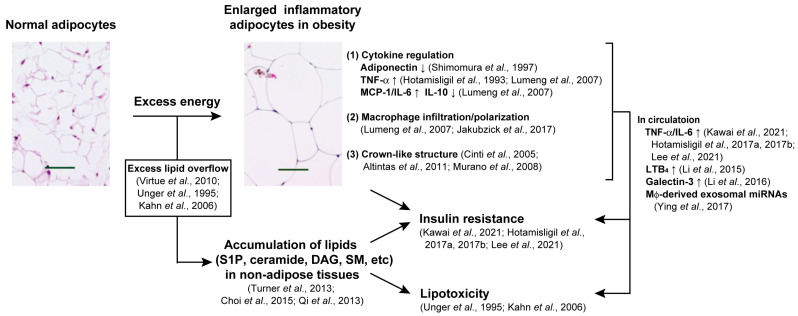
Systemic insulin resistance and lipotoxic damage caused by excessive lipid accumulation in both adipose and nonadipose tissues [27,28,29,30,31,32,33,34,35,36,37,38,39,40,41,42,43,44,45,46,47].

Several mechanisms have been proposed to explain the association between obesity and metabolic disease. Adipose tissues are essential for regulating lipid and glucose homeostasis, and patients with lipoatrophic diabetes, who tend to lack adipose tissues, exhibit severe insulin resistance, hyperglycemia, dyslipidemia, and hepatic steatosis [26]. If adipocytes cannot store highly hydrophobic (and, thus, cytotoxic) triglyceride, excessive fat leaks into the blood as non-esterified fatty acids (NEFA). The NEFA in the circulation overflows into other tissues, such as the liver, skeletal muscle, heart, and pancreas, via adipose tissue expandability/expansion [27], and can confer systemic insulin resistance and organ damage, that is, lipotoxicity (Figure 1) [28,29].

In experimental animals fed HFDs, the chemical composition of fats and the extent of insulin resistance varied between tissues. Diacylglycerol and short-chain fatty acid-type ceramides accumulate in the liver and skeletal muscle. In contrast, short-type ceramides and sphingomyelin (and diacylglycerol after 16 weeks of HFDs) accumulate in the adipose tissue, which may confer organ-specific insulin resistance [30]. In addition, a lack of adipose tissue causes the loss of “beneficial” hormones, such as leptin and adiponectin. The exacerbation of insulin resistance is partially recovered by supplementation with these hormones in patients with lipoatrophic diabetes [48]. Thus, obesity inhibits the accumulation of triglycerides in adipocytes and decreases adiponectin [31]. Moreover, mitochondrial mass and adipocyte function are suppressed in obese mice [49] and humans [50]. However, whether adipocytes are damaged by mitochondrial dysfunction remains unclear.

### 2.3. Adipose Tissue Inflammation

Adipose tissue inflammation was first noticed by tumor necrosis factor α (TNFα) expression in the adipose tissue and stromal vascular fraction of obese animals [32]. Cytokines such as interleukin (IL)-6, IL-8, IL-10, and granulocyte colony-stimulating factor (G-CSF) were reported to be observed in adipocytes [32,33,51]. Monocyte chemoattractant protein-1 (MCP-1) is highly expressed in obese adipose tissue [33] and induces macrophage infiltration into adipose tissue, as well as insulin resistance [52]. These findings imply that MCP-1 secreted by obese adipocytes induces macrophage infiltration into the adipose tissue, which in turn causes adipose tissue inflammation, insulin resistance, and hepatic steatosis (Figure 1). Additionally, most of the adipose tissues from obese mice had classically activated (M1) macrophages that produced various pro-inflammatory cytokines, such as IL-1, IL-6, and TNFα, and reactive oxygen species. In contrast, most of the tissues from lean mice had alternatively activated anti-inflammatory (M2) macrophages [34,35]. A crown-like structure composed of dead or dying hypertrophic adipocytes surrounded by macrophages is a hallmark of chronic inflammation in adipose tissue [36,37,38]. These findings suggest that obesity induces adipose tissue inflammation and insulin resistance (Figure 1) [39,40,41,42].

In obese humans and mice, macrophages accumulate more in VAT than in SAT [37,38,53]. Therefore, inflammation in VAT may be caused by the onset of metabolic disorders [54], although several researchers have observed the importance of inflammation in SAT [55]. Adipose tissue inflammation may induce systemic insulin resistance via the circulating cytokines (e.g., TNFα and IL-6) secreted by M1 macrophages that attenuate insulin signaling in the liver and the skeletal muscle [39,40,41,42]. Leukotriene B_4_ (LTB_4_) and galectin-3 have been postulated as candidates linking adipose tissue inflammation and insulin resistance [43,44]. Furthermore, macrophage-derived exosomal miRNAs impair insulin action in the liver and skeletal muscles [45]. By contrast, obesity can induce M1 macrophage accumulation in the liver, skeletal muscles, and pancreas, which may cause insulin resistance (Figure 1) [42].

The fundamental question is whether obesity-induced adipose tissue inflammation is comparable to the common chronic inflammation associated with infection and cancer. Ordinary chronic inflammation is accompanied by reduced appetite and increased energy expenditure, leading to weight loss and, in the worst cases, cachexia. In contrast, inflammation in obesity does not have this effect [41]. Kratz et al. demonstrated that CD274, CD38, and CD319 were expressed in classically activated M1 macrophages isolated from patients with cystic fibrosis. In contrast, they were hardly observed in metabolically activated M1 macrophages isolated from the adipose tissue of patients with obesity [56]. The expression levels of TNFα and IL-6 were much lower in metabolically activated M1 macrophages than those in classically activated M1 macrophages [56]. Furthermore, obesity-induced lysosomal-dependent lipid metabolism was not observed in classically activated M1 macrophages [57]. Recent studies have revealed that the population of adipose tissue macrophages is more complex than previously expected [58,59]. Thus, although common processes may exist, the inflammation in obese adipose tissues may differ from classical inflammation.

These findings may be related to the failure of multiple anti-inflammatory treatments to ameliorate obesity-induced metabolic disorders. In clinical trials using TNFα-targeted drugs that are effective against rheumatoid arthritis, a few were slightly effective in improving insulin sensitivity [60], while others were not [61,62]. In patients with T2DM, the blockade of IL-1 receptor signaling improves glycemic control and the ability to secrete insulin, but not insulin resistance itself, as evaluated by insulin-regulated gene expression in skeletal muscle and serum adiponectin levels [63]. Although the administration of high doses of salicylate has been shown to improve blood glucose levels in mice [64], no success has been observed in treatments that target adipose tissue inflammation in patients with obese T2DM [39,42].

## 3. S1P and S1P Receptors

**Figure 2:**
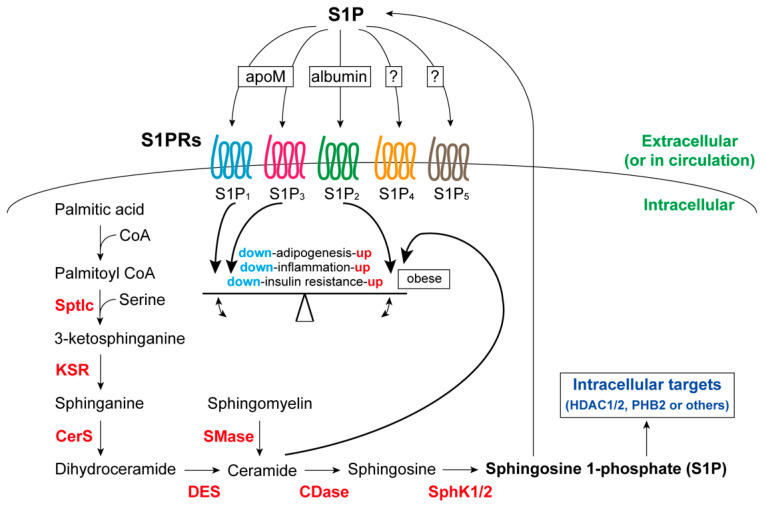
Sphingosine 1-phosphate (S1P) production and associated cell surface receptor signaling that regulates adipose function. Ceramide is produced from sphingomyelin by sphingomyelinase (SMase) or from palmitoyl-CoA and serine via serine palmitoyltransferase (Sptlc), 3-ketosphinganine reductase (KSR), ceramide synthase (CerS), and dihydroceramide desaturase (DES). Ceramide is converted to sphingosine by ceramidase (CDase) and S1P by SphK1/2. S1P acts through intracellular targets (HDAC1/2, PHB2, etc.) or is secreted extracellularly (e.g., in circulation) to affect cell surface S1P receptors (S1P_1_–S1P_5_). S1P_1/3_ agonists and S1P_2_ agonists/ceramide have opposing actions in terms of adipose function: the former prevents obesity and associated adipogenesis, adipocyte inflammation, and insulin resistance, whereas the latter rather promotes such conditions [1,2].

S1P was first described in 1991 as a growth factor derived from membrane sphingolipids [65]. Sphingomyelinase converts sphingomyelin to ceramide, which is metabolized to sphingosine by ceramidase (Figure 2) [66]. Ceramides are synthesized from serine and palmitoyl-CoA through the activities of enzymes such as serine palmitoyltransferase (serine palmitoyltransferase long-chain base subunit: Sptlc) and ceramide synthase (CerS) (Figure 2) [67]. Obesity causes an excessive flow of saturated fatty acids into adipocytes and other tissues, along with the accumulation of ceramide, a key lipotoxic player [68]. Sphingosine is phosphorylated by sphingosine kinase 1 (SphK1) and SphK2, which have similar catalytic properties but differ in subcellular localization and tissue-specific expression [66]. SphK1 is cytosolic and translocates to the plasma membrane or extracellularly upon activation [67], whereas SphK2 is located in the endoplasmic reticulum, mitochondria, and nucleus [69]. S1P is secreted as a paracrine or endocrine molecule and is an intracellular secondary messenger. Circulating (serum) S1P levels are approximately 500 nM in humans [70], and extracellular S1P binds to high-density lipoproteins via apolipoprotein M (apoM) and albumin (Figure 2) [71]. S1P are degraded by S1P lyase (SPL) or S1P phosphatases (SPP1 and SPP2) [72].

S1P participates in various cellular signaling pathways, including those involved in cell survival, proliferation, migration, and differentiation in multiple organs, through five cognate S1PRs: S1P_1_–S1P_5_ (Figure 2) [72,73]. S1P_1_–S1P_3_ are expressed in various cell types, whereas S1P_4_ and S1P_5_ are expressed exclusively in lymphocytes and dendritic cells, respectively [74]. The characteristics and roles of S1PRs have been described in many reviews [72,73]. Therefore, we only cover the following three issues: First, the role of S1P_2_ in inflammation, especially in macrophage function, is complex, and S1P_2_ has been proposed to suppress [75] or promote [76] macrophage activity. Similarly, the opposite relationship was observed between S1P_3_ and inflammation [77,78], probably attributable to differences in inflammation and the surrounding environment. Second, these receptors may act cooperatively and antagonistically [79,80], underscoring the diverse physiological actions of S1P. Therefore, when we discuss S1P actions, identification of the S1P receptor subtypes involved becomes indispensable. Third, extracellularly released S1P often acts against intracellular S1P levels. Activation of SphK1 in response to pro-inflammatory stimuli may induce M1 macrophage formation when S1P_1_ activation triggers anti-inflammatory responses [81]. The intracellular S1P targets (especially those important in glucose homeostasis) remain to be clarified (Figure 2). However, S1P may directly bind to and inhibit histone deacetylase 1/2 (HDAC1/2) [82] or bind to prohibitin 2 (PHB2) to regulate cytochrome *c* oxidase assembly and mitochondrial respiration [83].

Clinical applications focusing on the potent action of S1P began in the 2010s. FTY720 (Fingolimod™) was the first clinically approved S1P signal modulator for treating multiple sclerosis [84]. FTY720 binds to and activates all S1PRs, except S1P_2_, but specifically downregulates S1P_1_ in lymphocytes [74,85]. Furthermore, FTY720 suppresses CerS activity but activates ceramide synthesis in cultured cells under certain circumstances [86]. Next-generation S1PR modulators with fewer adverse effects and receptor specificities, including mocravimod (S1P_1/4/5_ agonist), ozanimod (S1P_1/5_ agonist), etrasimod (S1P_1/4/5_ agonist), amiselimod (S1P_1_ agonist), ponesimod (S1P_1/4/5_ agonist), siponimod (S1P_1/5_ agonist), and ceralifimod (S1P_1/5_ agonist), were developed and investigated for clinical application in diseases other than multiple sclerosis, including inflammatory bowel disease, psoriasis, atopic dermatitis, rheumatoid arthritis, systemic lupus erythematosus, and certain cancers [87]. In the laboratory, SEW-2871 has been used as a S1P_1_ agonist, VPC-23019 as a S1P_1/3_ antagonist, JTE-013 as a S1P_2_ antagonist, and CYM-50358 as a S1P_4_ antagonist [1,2].

Several drugs that regulate S1P signaling have been developed in addition to S1PR agonists/modulators. Several SphK inhibitors have been developed, including isoform-specific ones [88]. SPL inhibitors LX2931 and LX2932 have also been developed to treat rheumatoid arthritis [89].

## 4. Tissue-Specific Roles of S1P/S1PR in Insulin Resistance

Obesity-induced insulin resistance occurs in pancreatic β-cells and peripheral tissues such as the liver, adipose tissue, and skeletal muscle [90]. Genetic deletion of insulin receptors in murine adipose tissue, skeletal muscle, or both induces insulin resistance, but not diabetes [91,92], which contrasts with the liver-specific deletion of insulin receptors that causes severe insulin resistance and hyperglycemia [93]. Therefore, hepatic insulin resistance is considered more important in whole-body glucose metabolism. However, the roles of adipose tissues, especially in obese states, dominate. The levels of S1P/ceramide (diacylglycerol/sphingomyelin) and the activation status of SphK/S1PR have been investigated in the tissues and plasma of HFD-fed obese mice (Table 1). S1P and ceramide levels increased in the liver, adipose tissue, skeletal muscle, pancreas, and plasma [30,46], except for ceramide in the pancreas [47]. SphK1 and SphK2 can be activated in the liver [94,95], whereas only SphK1 is activated in adipose tissue [96] and skeletal muscle [94]. Information regarding the S1PR subtypes involved in obesity is limited; however, S1P_3_ is upregulated in the liver and adipose tissue [97]. Furthermore, S1P_1_ is upregulated, whereas S1P_3_ is downregulated in the skeletal muscle [98].

### 4.1. Liver

The effects of S1P/S1PR signal modification on the livers of experimental mice are summarized in Table 2. As observed in patients with NAFLD, hepatic insulin resistance is usually associated with hepatic steatosis, resulting from excessive incorporation of fatty acids into the liver and upregulation of de novo lipogenesis. Inhibition of insulin receptor kinase by protein kinase C activation induced by diacylglycerol and reduced expression of insulin receptor substrate 2 (IRS2), a signaling molecule downstream of insulin receptor kinase, could cause hepatic insulin resistance [99]. The paradoxical state of elevated hepatic de novo lipogenesis in the presence of insulin resistance is known as “selective hepatic insulin resistance” [100].

Insulin resistance and NAFLD form a vicious cycle by which they exacerbate each other. NAFLD can be accompanied by inflammation and fibrosis (nonalcoholic steatohepatitis, NASH), which may progress irreversibly to cirrhosis and hepatocellular carcinoma. These pathogeneses, which begin with insulin resistance, have been extensively studied, and the involvement of SphK, S1P, and S1PRs has been proposed [94,99,101]. The sources of sphingolipids and ceramides are long-chain saturated fatty acids deposited in the liver; hence, the amount of ceramide is upregulated in the liver during hepatic steatosis [102]. Ceramide can affect insulin-stimulated Akt activation and subsequent glucose uptake in the liver and skeletal muscle [103], which is another cause of insulin resistance. In HFD-fed obese mouse models, reduced C16:0 ceramide by antisense oligonucleotide knockdown of ceramide synthase 6 (CerS6) improved glucose resistance and insulin sensitivity [104]. S1P, a terminal metabolite of ceramide whose content is increased in the livers of patients with NAFLD [105], induces insulin resistance in rat hepatocytes [93]. Injecting the S1P_2_ antagonist JTE-013 daily for seven consecutive days lowered blood glucose levels and increased phosphorylated Akt levels in the liver fractions of HFD-fed obese mice [106]. Hepatic glucose intolerance and insulin resistance have been observed in hepatocyte-specific SphK2-knockout (SphK2^−/−^) mice [107]. Interestingly, the addition of ARN14974 (an inhibitor of acid ceramidase), but not S1P, restored insulin resistance in SphK2-null Huh7 hepatic cell lines, suggesting that insulin resistance in hepatocyte-specific SphK1^−/−^ mice is associated with the accumulation of sphingosine rather than decreased S1P production [107]. In contrast, the adenoviral overexpression of SphK1 in the liver improved glucose tolerance and hepatic steatosis in KK/Ay diabetic mice [108].

Additionally, the reduction in hepatic steatosis and improvement in insulin signals in the livers of HFD-induced obese mice were accompanied by reduced adiposity induced by adipocyte-specific genetic deletion of Sptlc2 and treatment with its potent inhibitor, “myriocin” [97]. Systemic genetic deletion or pharmacological inhibition (with a specific inhibitor “5C”) of SphK1 reduced hepatic steatosis and upregulated Akt activity in the livers of HFD-induced obese mice [96]. Systemic genetic deletion of S1P_3_ exacerbated HFD-induced hepatic steatosis [109]. In contrast, oral administration of JTE-013 (an S1P_2_ antagonist) and SEW-2871 (an S1P_1_ agonist) failed to alleviate hepatic steatosis [2]. In HFD-induced obese mice, the levels of plasma S1P and its carrier, apoM, were upregulated. Deterioration of insulin resistance was observed in apoM^−/−^ mice, and improved insulin resistance was observed in apoM-overexpressing mice [110].

**Table 2:** Effects of modification of the SphK1/S1P/S1PR axis on insulin actions in the liver.

Intervention	Applied Mice	Glc. Tol.	Ins. Res.	Steatosis	Insulin Action	Ref.
CerS6 ASO knockdown	HFD obese or ob/ob	Improved	Improved	Improved	NE	[104]
JTE-013 (S1P_2_ blocker)	HFD NZ obese	Improved	NE	NE	p-Akt↑;GSK-3b↑; glycogen synthesis↑	[106]
Hepatocyte-specific SphK2^−/−^	HFD SphK2^−/−^	Impaired	Impaired	Impaired	p-Akt↓; hepatic glucose production↑	[107]
Ad-SphK1 overexpression	HFD KK/Ay	Improved	NE	Improved	p-Akt↑; GSK-3b↑	[108]
Adipocyte-specific Sptlc2^−/−^	HFD Sptlc2^−/−^	Improved	Improved	Improved	hepatic glucose production →	[97]
Myriocin (Sptlc2 inhibitor)	HFD obese	Improved	Improved	Improved	NE	[97]
SphK1^−/−^	HFD SphK1^−/−^	Improved	Improved	Improved	p-Akt ↑	[96]
5C (SphK1 inhibitor)	HFD obese	Improved	Improved	NE	p-Akt ↑	[96]
S1P_3_^−/−^	HFD S1P_3_^−/−^	Impaired	Impaired	Impaired	NE	[109]
JTE-013 or SEW-2871	HFD obese	Improved	NE	→	NE	[2]
ApoM^−/−^	HFD obese	Impaired	Impaired	NE	p-Akt ↓	[110]
Ad-apoM overexpression	HFD obese	Improved	Improved	NE	p-Akt ↑	[110]

Glc. Tol., glucose tolerance; Ins. Res., insulin resistance; CerS6, ceramide synthase 6; ASO, antisense oligonucleotide; GSK, glycogen synthetase kinase; NE, not examined; Ad, adenoviral; ↑, upregulated; ↓, downregulated; →, no change. The reference numbers are in parentheses.

### 4.2. Adipose Tissue

Table 3 summarizes the impact of S1P/S1PR signal modification on adipose tissue. SphK1/2 expression and S1P content were upregulated at the terminal differentiation stage of 3T3-L1 adipocytes, and the suppression of SphK1 (but not SphK2) attenuated their differentiation into mature adipocytes [111]. SphK1 mRNA levels were higher in adipose tissues from ob/ob mice than in those from control mice [96]. Administration of S1P to 3T3-L1 adipocytes attenuated their adipogenic differentiation [112], and S1P inhibited adipogenic differentiation and enhanced the osteogenic differentiation of mesenchymal stem cells [113]. These contradictory results suggest that adipocyte differentiation requires specific amounts of S1P, and excessive S1P levels may inhibit differentiation.

Compared to the corresponding wild-type mice, HFD-fed SphK1^−/−^ mice exhibited increased adipose tissue weight; smaller adipocyte size; increased expression of adipocyte markers; and improved glucose tolerance, insulin sensitivity, and adipose tissue inflammation [96]. Intraperitoneal injection of 5C (an SphK1 inhibitor) improved glucose tolerance, insulin resistance, and adipose tissue inflammation in HFD-fed mice [96]. Our group has demonstrated that the application of S1P and JTE-013 downregulates adipogenesis. In contrast, the application of VPC23019 (an S1P_1/3_ antagonist) upregulates the adipogenic differentiation of 3T3-L1 and F442A adipocytes [1]. These results suggest that S1P_1_ signaling suppresses adipogenic differentiation, whereas S1P_2_ accelerates it, and S1P_2_ dominates the overall functions of S1P. Regarding preadipocyte proliferation, the blockade of S1P_1_ signaling by VPC-23019 inhibited the proliferation of 3T3-L1 and F442A preadipocytes; in contrast, the blockade of S1P_2_ signaling by JTE-013 accelerated it [1]. These findings are consistent with the idea that S1P_1_ signals accelerate preadipocyte proliferation, whereas S1P_2_ signals inhibit it, and S1P_1_ governs the overall S1P actions. We further investigated the effect of S1P_2_ deletion on adipose tissue and glucose metabolism in S1P_2_-knockout (S1P_2_^−/−^) mice [116]. S1P_2_^−/−^ mice fed a normal diet had lower body weights and smaller epididymal adipocytes than those in wild-type mice, while displaying glucose tolerance and adipocyte marker gene expression similar to wild-type mice [1]. However, after four weeks of HFD feeding, S1P_2_^−/−^ mice exhibited much smaller adipocytes with improved glucose intolerance/insulin sensitivity, accompanied by reduced crown-like structures and improved M1/M2 macrophage polarization in adipose tissue sections [1]. Consequently, we speculate that S1P_2_ deletion accelerates preadipocyte proliferation and suppresses adipogenic differentiation, which may induce adipocyte hyperplasia and prevent glucose intolerance, insulin resistance, and adipose tissue inflammation. Additionally, oral administration of JTE-013 to ob/ob mice for four weeks reduced body weight and improved glucose tolerance and insulin sensitivity [1].

Administration of JTE-013 or SEW-2871 for 12 weeks reduced body weight gain and adipocyte size in both epididymal and inguinal adipose tissues of ob/ob mice [2], and improved glucose intolerance and inflammation in epididymal adipose tissue (but not hepatic steatosis); however, all SEW-2871 effects were canceled by co-administration with VPC-23019 [2]. Consistent with the results in HFD-fed S1P_2_^−/−^ mice [1], preventing adipose tissue inflammation and glucose intolerance using JTE-013 and SEW-2871 was attributed to reducing adipocyte size rather than weight loss. Moreover, S1P_3_-knockout (S1P_3_^−/−^) mice [117] exhibited phenotypes opposite to those of S1P_2_^−/−^ mice, that is, impaired glucose intolerance, adipose tissue inflammation, and reduced Adipoq mRNA expression in the adipose tissue [109]. Conversely, the adenoviral overexpression of S1P_2_ in 3T3-L1 adipocytes inhibited adipogenic differentiation [118]. Therefore, the discrepancy in the role(s) of S1P_2_ in adipogenic differentiation may be attributed to differences between constitutive S1P_2_ deletion in mice [1] and transient exogenous S1P_2_ overexpression in cell lines [118]. FTY720 administration in HFD-fed obese mice caused decreased body weight gain, improved glucose tolerance, and adipose tissue inflammation [114], and prevented body weight and fat weight gain [105]. FTY720 reduces adipocyte size, inhibits adipogenesis, and promotes lipolysis via unknown mechanisms [115]. However, if FTY720 acts as an S1P_1/3/4/5_ agonist in adipocytes, these findings may be consistent with our results.

Ceramide accumulation in VAT appeared to correlate with metabolic disorders in mice [97]. In HFD-induced obese mice, adipocyte-specific genetic deletion of Sptlc2 or treatment with its inhibitor myriocin reduced the levels of adipose sphingolipids and improved adipocyte hypertrophy, systemic glucose tolerance, insulin resistance, and adipose tissue inflammation [97]. Myeloid-specific Sptlc2 deletions exhibited reduced Sptlc2 expression and myeloid sphingolipid levels. However, they did not affect body or fat weights, glucose tolerance, insulin sensitivity, or adipose tissue morphology and inflammation; therefore, macrophage sphingolipids do not contribute to the adipose phenotypes that result from global inhibition of Sptlc2 [97].

The regulatory role of S1P in inflammation may be due to either its direct effects on macrophages or its indirect effects on adipocytes; however, previous in vivo studies have not fully addressed this question. Several attempts to improve metabolic abnormalities by regulating adipose tissue inflammation have been unsuccessful, and studies employing mice lacking each of the S1PRs in macrophage- or adipocyte-specific manners are anticipated.

### 4.3. Skeletal Muscle

The effects of S1P/S1PR signal modification in the skeletal muscle, the most abundant tissue, are summarized in Table 4. The roles of sphingolipids in insulin signaling in the skeletal muscle are simpler than those in the liver and adipose tissue; ceramide impairs insulin signals in skeletal muscle, but S1P enhances them [94]. Palmitate induces ceramide generation, thereby preventing insulin-induced Akt activation and glycogen synthesis [119], whereas S1P enhances basal and insulin-induced glucose uptake via S1P_2_ in C2C12 myoblasts [120]. Dexamethasone treatment of C2C12 cells induces atrophy accompanied by reduced SphK1 phosphorylation (activation) and reduced intracellular S1P production while maintaining extracellular S1P production and upregulating cell-surface S1P_2_ expression, suggesting pathophysiological roles of S1P/S1PR in skeletal muscle [121]. Global transgenic overexpression of SphK1 using the universal CAG promoter in HFD-fed mice improved insulin resistance in the whole body and skeletal muscle, which was associated with decreased intramuscular ceramide accumulation (but not S1P accumulation) compared to that in their respective wild-type littermates [122]. FTY720 administration to HFD-fed mice prevented ceramide accumulation in the skeletal muscle and insulin resistance in the whole body or skeletal muscle without downregulating any S1PR [98]; therefore, the involvement of S1P/S1PR remains unknown. Skeletal muscle secretes IL-6, which can improve glucose tolerance via several mechanisms [123]. Excessive exogenous palmitate induces SphK1 and IL-6 mRNA expression via S1P_3_ in mouse myotubes, but not in adipocytes [124]. In addition, adipocyte-specific deletion of Sptlc2, systemic deletion of SphK1, and treatment with 5C improved glucose tolerance and insulin sensitivity in the skeletal muscle [96,97]. Akt phosphorylation levels in the skeletal muscle were attenuated in apoM^−/−^ mice compared to those in wild-type mice, suggesting the involvement of skeletal muscle in systemic glucose intolerance [110]. Compared to the liver and adipose tissue, our knowledge regarding the roles of the SphK/S1P/S1PR axis in the skeletal muscle (and pancreatic β-cells) is rather limited.

### 4.4. Pancreatic β-Cells

The effects of S1P/S1PR signal modification on β-cells are summarized in Table 5. Obesity-induced insulin resistance forces pancreatic β-cells to secrete more insulin, which is highly stressful to β-cells, and obesity-induced lipotoxicity causes β-cell failure [90]. Although secreted insulin can exert a negative feedback effect on β-cells through insulin receptors and/or insulin-like growth factor-1 receptors [125], a deficiency in IRS-2 causes β-cell dysfunction, indicating that the insulin signal is essential for the survival and maintenance of β-cells [126]. The exposure of cultured rat primary islet cells to palmitate caused ceramide accumulation, β-cell apoptosis, and reduced insulin secretion [127]. SphK1 knockdown in rat insulinoma INS-1 832/13 cells reduced insulin synthesis and secretion, whereas SphK1 overexpression restored them [128]. Glucose increases the S1P content by activating SphK2 in mouse insulinoma MIN6 cells and mouse pancreatic islet cells, whereas SphK2 knockdown reduces glucose-stimulated insulin secretion [129]. Furthermore, treating mice with an SphK inhibitor induces glucose intolerance and decreases plasma insulin levels [129].

HFD-fed SphK1^−/−^ mice exhibited more evident diabetic conditions, including reduced plasma insulin levels associated with reduced β-cell mass and increased β-cell apoptosis, than the respective wild-type mice [47]. These results imply that S1P increases insulin synthesis and secretion, although the relationship is more complex. Evidence suggests that intracellular S1P impairs β-cell function and survival, while extracellular S1P protects β-cells [101]. Oral administration of FTY720 prevents the development of diabetes by increasing the proliferation of β-cells without affecting insulin sensitivity, which is mediated by S1P_1/3_ receptors [130]. Conversely, streptozotocin-induced apoptosis of β-cells was attenuated in S1P_2_^−/−^ mice, implying that S1P_2_ signaling interferes with β-cell survival [131].

### 4.5. S1P in the Circulation

The physiological roles of S1P in circulation are not fully understood. S1P binds stably to apoM, primarily associated with HDL, or binds unstably to albumin. ApoM-bound S1P activates S1P_1/3_ to protect against IgA nephropathy, whereas albumin-bound S1P activates S1P_2_ to exert deteriorating effects [132]. Therefore, the physiological roles of S1P in circulation may depend on its carrier protein. The APOM polymorphism, rather than serum apoM levels, appears to be correlated with the risk of T2DM [133]. However, serum apoM levels were inversely associated with BMI and the insulin resistance index [110]. ApoM^−/−^ mice exhibited deteriorated insulin resistance, whereas apoM-overexpressing mice showed improvements in insulin resistance, presumably through the activation of S1P_1/3_ signaling [110].

## 5. Concluding Remarks

Several studies have emphasized the regulatory roles of SphK, S1P, and S1PR signaling in systemic and local insulin sensitivity. Although adipose-tissue-specific deletion of insulin receptors has no effect on systemic glucose metabolism [91], obesity and the accompanying adipose tissue growth are the most important etiologies of insulin resistance. The onset of insulin resistance is linked to several alterations in adipokine secretion, lipotoxicity, and inflammation of adipose tissue (particularly VAT) accompanied by adipocyte hypertrophy (Figure 1). Reduced plasma and adipose tissue S1P levels in SphK1^−/−^ mice improved insulin resistance, which is associated with reducing adipocyte hypertrophy and inflammation in adipose tissue [96]. Our observations that either S1P_1_ activation or S1P_2_ blockage ameliorated adipocyte hypertrophy, glucose intolerance, and inflammation in the VAT of obese mice [1,2] were consistent with previous results showing that apoM-carrying S1P worked through S1P_1/3_ to improve insulin resistance, whereas albumin-carrying S1P activated S1P_2_ to exacerbate it in obese adipocytes (Figure 2) [110,132] and the liver [106]. Hepatic insulin resistance is caused by the accumulation of ceramide and its metabolite, sphingosine [103,104,107]. The upregulation of ceramide interferes with insulin action, whereas S1P improves insulin resistance in C2C12 myoblasts [120]. However, the function of S1P signaling in regulating insulin sensitivity of skeletal muscles in vivo has not yet been thoroughly explained. As for pancreatic β-cells, the functions of the SphK/S1P/S1PR axis in insulin secretion and their survival are points of issue. Several studies have supported the idea that either the activation of S1P_1/3_ or the blockade of S1P_2_ is beneficial to adipose tissue, liver, and pancreatic β-cells in obese diabetic animals.

Several problems remain before the SphK/S1P/S1PR axis clinical application can begin. Because S1P exerts pleiotropic actions in any type of cell throughout the body, it is conceivable that S1P agonists or antagonists demonstrate unexpected actions beyond the expected actions on target targets. S1P_1/3_ agonists may affect carcinogenesis. In this regard, approved FTY720, which acts on ceramide synthesis and S1P_1,3,4,5_ as either agonist or antagonist depending on cell types is far from ideal, even though they effectively improve metabolic abnormalities induced by obesity in adipose tissue, skeletal muscles, and pancreatic β-cells with rather limited side effects. According to our study, a new class of S1P_1_-specific agonists, SEW-2871, and an S1P_2_ antagonist, JTE-013, could be used as therapeutic options for obese diabetes patients [1,2]. Because our studies mainly examined the effects of SEW-2871 and JTE-013 on adipocytes, their influences on the liver, skeletal muscle, and pancreatic β-cells should be carefully investigated.

In conclusion, the SphK/S1P/S1PR axis, which drastically regulates adipocyte function and glucose homeostasis, is a potential target for next-generation drugs against obesity and associated metabolic disorders.

## Figures and Tables

**Table 1:** Lipid levels and SphK/S1PR activation status in tissues of HFD-fed obese mice.

	Liver	Adipose Tissue	Skeletal Muscle	Pancreas	Plasma
S1P levels	↑ [46]	↑ [46]	↑ [46]	↑ [47]	↑ [46]
Ceramide levels	↑ [30,46]	↑ [30,46]	↑ [30,46]	→ [47]	↑ [46]
Diacylglycerol levels	↑ [30]	↑ [30]	↑ [30]	NE	NE
Sphingomyelin levels	↑ [30]	NE	NE	NE	NE
SphK activation	SphK1↑ [94]	SphK1↑ [96]	SphK1↑ [94]	NE	NE
	SphK1→/SphK2↑ [95]				
S1PR activation	S1P_3_↑ [97]	S1P_3_↑ [97]	S1P_1_↑/S1P_2_→/S1P_3_↓ [98]	NE	NE

NE, not examined; ↑, upregulated; ↓, downregulated; →, no change. The reference numbers are in parentheses.

**Table 3:** Effects of modification of the SphK1/S1P/S1PR axis on insulin actions in the adipose tissue.

Intervention	Applied Mice	BW	FW	Glc. Tol.	Ins. Res.	Size	Inflammation	Insulin Action	Ref.
SphK1^−/−^	HFD SphK1^−/−^	→	↑	Improved	Improved	↓	CLS ↓; M1/M2 ↓	glucose uptake↑	[96]
5C (SphK1 inhibitor)	HFD obese	NE	NE	Improved	Improved	NE	M1/M2 ↓	p-Akt↑	[96]
S1P_2_^−/−^	S1P_2_^−/−^	↓	↓	→	→	↓	M1/M2 →	NE	[1]
S1P_2_^−/−^	HFD S1P_2_^−/−^	→	↓	Improved	Improved	↓	CLS ↓; M1/M2 ↓	NE	[1]
JTE-013	ob/ob	↓	↓	Improved	Improved	↓	M1/M2 ↓	NE	[1,2]
SEW-2871 (S1P_1_ agonist)	ob/ob	↓	↓	Improved	NE	↓	M1/M2 ↓	NE	[2]
S1P_3_^−/−^	HFD S1P_3_^−/−^	→	↓	Impaired	Impaired	NE	CLS ↑; M1/M2 ↑	NE	[109]
FTY720	HFD obese	↓	NE	Improved	NE	NE	M1/M2 ↓	NE	[114]
FTY720	HFD obese	↓	↓	NE	NE	↓	NE	p-Akt↑; p-AMPK↑	[115]
Adipocyte-specific Sptlc2^−/−^	HFD Sptlc2^−/−^	↓	↓	Improved	Improved	↓	CLS ↓; M1/M2 ↓	glucose uptake↑	[97]
Myeloid-specific Sptlc2^−/−^	HFD Sptlc2^−/−^	→	→	→	→	→	CLS →	NE	[97]
Myriocin (Sptlc2 inhibitor)	HFD obese	↓	↓	Improved	Improved	↓	CLS ↓; M1/M2 ↓	NE	[97]

BW, body weight; FW, fat weight; Glc. Tol., glucose tolerance; Ins. Res., insulin resistance; NE, not examined; CLS, crown-like structure; M1/M2, M1 macrophage/M2 macrophage polarization; ↑, upregulated; ↓, downregulated; →, no change.

**Table 4:** Effects of modification of the SphK1/S1P/S1PR axis on insulin actions in the skeletal muscle.

Intervention	Applied mice	Glc. Tol.	Ins. Res.	Insulin Action	Ref.
SphK1 overexpression	HFD SphK1 TG	Improved	Improved	Glucose uptake↑	[122]
FTY720	HFD obese	Improved	NE	Glucose uptake↑; p-Akt↑	[98]
Adipocyte-specific Sptlc2^−/−^	HFD Sptlc2^−/−^	Improved	Improved	p-Akt↑	[97]
SphK1^−/−^	HFD SphK1^−/−^	Improved	Improved	p-Akt→	[96]
5C (SphK1 inhibitor)	HFD obese	Improved	Improved	p-Akt→	[96]
ApoM^−/−^	HFD obese	Impaired	Impaired	p-Akt↓	[110]

Glc. Tol., glucose tolerance; Ins. Res., insulin resistance; TG, transgenic; NE, not examined; ↑, upregulated; ↓, downregulated; →, no change.

**Table 5:** Effects of modification of the SphK1/S1P/S1PR axis on insulin actions in pancreatic β-cells.

Intervention	Applied Mice	BW	Glc. Tol.	Ins. Res.	Ins. Secretion	Survival	Ref.
SphK1^−/−^	HFD SphK1^−/−^	→	Impaired	→	↓	↓	[47]
FTY720	*db*/*db*	↑	Improved	→	↑	↑	[130]
S1P_2_^−/−^	STZ-induced diabetic	NE	NE	NE	↑	↑	[131]

Glc. Tol., glucose tolerance; Ins. Res., insulin resistance; STZ, streptozotocin; NE, not examined; ↑, upregulated; ↓, downregulated; →, no change.

## Data Availability

The data presented in this study are available on request from the corresponding author.

## References

[1] Kitada Y., Kajita K., Taguchi K., Mori I., Yamauchi M., Ikeda T., Kawashima M., Asano M., Kajita T., Ishizuka T. (2016). Blockade of Sphingosine 1-Phosphate Receptor 2 Signaling Attenuates High-Fat Diet-Induced Adipocyte Hypertrophy and Systemic Glucose Intolerance in Mice. Endocrinology.

[2] Asano M., Kajita K., Fuwa M., Kajita T., Mori I., Akahoshi N., Ishii I., Morita H. (2023). Opposing roles of sphingosine 1-phosphate receptors 1 and 2 in fat deposition and glucose tolerance in obese male mice. Endocrinology.

[3] Hong C.H., Ko M.S., Kim J.H., Cho H., Lee C.H., Yoon J.E., Yun J.Y., Baek I.J., Jang J.E., Lee S.E. (2022). Sphingosine 1-phosphate receptor 4 promotes nonalcoholic steatohepatitis by activating NLRP3 inflammasome. Cell. Mol. Gastroenterol. Hepatol..

[4] Kim J.Y., Garcia-Carbonell R., Yamachika S., Zhao P., Dhar D., Loomba R., Kaufman R.J., Saltiel A.R., Karin M. (2018). ER stress drives lipogenesis and steatohepatitis via caspase-2 activation of S1P. Cell.

[5] Liao C.Y., Barrow F., Venkatesan N., Nakao Y., Mauer A.S., Fredrickson G., Song M.J., Sehrawat T.S., Dasgupta D., Graham R.P. (2023). Modulating sphingosine 1-phosphate receptor signaling skews intrahepatic leukocytes and attenuates murine nonalcoholic steatohepatitis. Front. Immunol..

[6] Maceyka M., Spiegel S. (2014). Sphingolipid metabolites in inflammatory disease. Nature.

[7] Drexler Y., Molina J., Mitrofanova A., Fornoni A., Merscher S. (2021). Sphingosine-1-phosphate metabolism and signaling in kidney diseases. J. Am. Soc. Nephrol..

[8] Cartier A., Hla T. (2019). Sphingosine 1-phosphate: Lipid signaling in pathology and therapy. Science.

[9] Lundgren M., Svensson M., Lindmark S., Renström F., Ruge T., Eriksson J.W. (2007). Fat cell enlargement is an independent marker of insulin resistance and ‘hyperleptinaemia’. Diabetologia.

[10] Yamauchi T., Kamon J., Waki H., Murakami K., Motojima K., Komeda K., Ide T., Kubota N., Terauchi Y., Tobe K. (2001). The mechanisms by which both heterozygous peroxisome proliferator-activated receptor gamma (PPARgamma) deficiency and PPARgamma agonist improve insulin resistance. J. Biol. Chem..

[11] Matsuzawa Y., Shimomura I., Nakamura T., Keno Y., Tokunaga K. (1993). Pathophysiology and pathogenesis of visceral fat obesity. Ann. N. Y. Acad. Sci..

[12] Huang H., Song T.J., Li X., Hu L., He Q., Liu M., Lane M.D., Tang Q.Q. (2009). BMP signaling pathway is required for commitment of C3H10T1/2 pluripotent stem cells to the adipocyte lineage. Proc. Natl. Acad. Sci. USA.

[13] Smas C.M., Sul H.S. (1993). Pref-1, a protein containing EGF-like repeats, inhibits adipocyte differentiation. Cell.

[14] Rodeheffer M.S., Birsoy K., Friedman J.M. (2008). Identification of white adipocyte progenitor cells in vivo. Cell.

[15] Berry R., Rodeheffer M.S. (2013). Characterization of the adipocyte cellular lineage in vivo. Nat. Cell Biol..

[16] Taguchi K., Kajita K., Kitada Y., Fuwa M., Asano M., Ikeda T., Kajita T., Ishizuka T., Kojima I., Morita H. (2020). Role of small proliferative adipocytes: Possible beige cell progenitors. J. Endocrinol..

[17] Rosen E.D., Walkey C.J., Puigserver P., Spiegelman B.M. (2000). Transcriptional regulation of adipogenesis. Genes Dev..

[18] Matsuzawa Y., Shimomura I., Nakamura T., Keno Y., Kotani K., Tokunaga K. (1995). Pathophysiology and pathogenesis of visceral fat obesity. Obes. Res..

[19] Chau Y.Y., Bandiera R., Serrels A., Martínez-Estrada O.M., Qing W., Lee M., Slight J., Thornburn A., Berry R., McHaffie S. (2014). Visceral and subcutaneous fat have different origins and evidence supports a mesothelial source. Nat. Cell Biol..

[20] Wang Q.A., Tao C., Gupta R.K., Scherer P.E. (2013). Tracking adipogenesis during white adipose tissue development, expansion and regeneration. Nat. Med..

[21] Schwalie P.C., Dong H., Zachara M., Russeil J., Alpern D., Akchiche N., Caprara C., Sun W., Schlaudraff K.U., Soldati G. (2018). A stromal cell population that inhibits adipogenesis in mammalian fat depots. Nature.

[22] Hepler C., Shan B., Zhang Q., Henry G.H., Shao M., Vishvanath L., Ghaben A.L., Mobley A.B., Strand D., Hon G.C. (2018). Identification of functionally distinct fibro-inflammatory and adipogenic stromal subpopulations in visceral adipose tissue of adult mice. Elife.

[23] Ghaben A.L., Scherer P.E. (2019). Adipogenesis and metabolic health. Nat. Rev. Mol. Cell Biol..

[24] Zhao G.N., Tian Z.W., Tian T., Zhu Z.P., Zhao W.J., Tian H., Cheng X., Hu F.J., Hu M.L., Tian S. (2021). TMBIM1 is an inhibitor of adipogenesis and its depletion promotes adipocyte hyperplasia and improves obesity-related metabolic disease. Cell Metab..

[25] Frank A.P., de Souza Santos R., Palmer B.F., Clegg D.J. (2019). Determinants of body fat distribution in humans may provide insight about obesity-related health risks. J. Lipid Res..

[26] Hussain I., Garg A. (2016). Lipodystrophy Syndromes. Endocrinol. Metab. Clin. N. Am..

[27] Virtue S., Vidal-Puig A. (2010). Adipose tissue expandability, lipotoxicity and the metabolic syndrome--an allostatic perspective. Biochim. Biophys. Acta.

[28] Unger R.H. (1995). Lipotoxicity in the pathogenesis of obesity-dependent NIDDM. Genetic and clinical implications. Diabetes.

[29] Kahn S.E., Hull R.L., Utzschneider K.M. (2006). Mechanisms linking obesity to insulin resistance and type 2 diabetes. Nature.

[30] Turner N., Kowalski G.M., Leslie S.J., Risis S., Yang C., Lee-Young R.S., Babb J.R., Meikle P.J., Lancaster G.I., Henstridge D.C. (2013). Distinct patterns of tissue-specific lipid accumulation during the induction of insulin resistance in mice by high-fat feeding. Diabetologia.

[31] Poulain-Godefroy O., Lecoeur C., Pattou F., Frühbeck G., Froguel P. (2008). Inflammation is associated with a decrease of lipogenic factors in omental fat in women. Am. J. Physiol. Regul. Integr. Comp. Physiol..

[32] Hotamisligil G.S., Shargill N.S., Spiegelman B.M. (1993). Adipose expression of tumor necrosis factor-alpha: Direct role in obesity-linked insulin resistance. Science.

[33] Kern P.A., Ranganathan S., Li C., Wood L., Ranganathan G. (2001). Adipose tissue tumor necrosis factor and interleukin-6 expression in human obesity and insulin resistance. Am. J. Physiol. Endocrinol. Metab..

[34] Lumeng C.N., Bodzin J.L., Saltiel A.R. (2007). Obesity induces a phenotypic switch in adipose tissue macrophage polarization. J. Clin. Investig..

[35] Jakubzick C.V., Randolph G.J., Henson P.M. (2017). Monocyte differentiation and antigen-presenting functions. Nat. Rev. Immunol..

[36] Cinti S., Mitchell G., Barbatelli G., Murano I., Ceresi E., Faloia E., Wang S., Fortier M., Greenberg A.S., Obin M.S. (2005). Adipocyte death defines macrophage localization and function in adipose tissue of obese mice and humans. J. Lipid Res..

[37] Altintas M.M., Azad A., Nayer B., Contreras G., Zaias J., Faul C., Reiser J., Nayer A. (2011). Mast cells, macrophages, and crown-like structures distinguish subcutaneous from visceral fat in mice. J. Lipid Res..

[38] Murano I., Barbatelli G., Parisani V., Latini C., Muzzonigro G., Castellucci M., Cinti S. (2008). Dead adipocytes, detected as crown-like structures, are prevalent in visceral fat depots of genetically obese mice. J. Lipid Res..

[39] Kawai T., Autieri M.V., Scalia R. (2021). Adipose tissue inflammation and metabolic dysfunction in obesity. Am. J. Physiol. Cell Physiol..

[40] Hotamisligil G.S. (2017). Inflammation, metaflammation and immunometabolic disorders. Nature.

[41] Hotamisligil G.S. (2017). Foundations of Immunometabolism and Implications for Metabolic Health and Disease. Immunity.

[42] Lee Y.S., Olefsky J. (2021). Chronic tissue inflammation and metabolic disease. Genes Dev..

[43] Li P., Oh D.Y., Bandyopadhyay G., Lagakos W.S., Talukdar S., Osborn O., Johnson A., Chung H., Maris M., Ofrecio J.M. (2015). LTB4 promotes insulin resistance in obese mice by acting on macrophages, hepatocytes and myocytes. Nat. Med..

[44] Li P., Liu S., Lu M., Bandyopadhyay G., Oh D., Imamura T., Johnson A.M.F., Sears D., Shen Z., Cui B. (2016). Hematopoietic-derived galectin-3 causes cellular and systemic insulin resistance. Cell.

[45] Ying W., Riopel M., Bandyopadhyay G., Dong Y., Birmingham A., Seo J.B., Ofrecio J.M., Wollam J., Hernandez-Carretero A., Fu W. (2017). Adipose tissue macrophage-derived exosomal miRNAs can modulate in vivo and in vitro insulin sensitivity. Cell.

[46] Choi S., Snider A.J. (2015). Sphingolipids in high fat diet and obesity-related diseases. Mediators Inflamm..

[47] Qi Y., Chen J., Lay A., Don A., Vadas M., Xia P. (2013). Loss of sphingosine kinase 1 predisposes to the onset of diabetes via promoting pancreatic β-cell death in diet-induced obese mice. FASEB J..

[48] Shimomura I., Hammer R.E., Ikemoto S., Brown M.S., Goldstein J.L. (1999). Leptin reverses insulin resistance and diabetes mellitus in mice with congenital lipodystrophy. Nature.

[49] Wilson-Fritch L., Nicoloro S., Chouinard M., Lazar M.A., Chui P.C., Leszyk J., Straubhaar J., Czech M.P., Corvera S. (2004). Mitochondrial remodeling in adipose tissue associated with obesity and treatment with rosiglitazone. J. Clin. Investig..

[50] Pietiläinen K.H., Naukkarinen J., Rissanen A., Saharinen J., Ellonen P., Keränen H., Suomalainen A., Götz A., Suortti T., Yki-Järvinen H. (2008). Global transcript profiles of fat in monozygotic twins discordant for BMI: Pathways behind acquired obesity. PLoS Med..

[51] Maachi M., Piéroni L., Bruckert E., Jardel C., Fellahi S., Hainque B., Capeau J., Bastard J.P. (2004). Systemic low-grade inflammation is related to both circulating and adipose tissue TNFalpha, leptin and IL-6 levels in obese women. Int. J. Obes. Relat. Metab. Disord..

[52] Kanda H., Tateya S., Tamori Y., Kotani K., Hiasa K., Kitazawa R., Kitazawa S., Miyachi H., Maeda S., Egashira K. (2006). MCP-1 contributes to macrophage infiltration into adipose tissue, insulin resistance, and hepatic steatosis in obesity. J. Clin. Investig..

[53] Cancello R., Tordjman J., Poitou C., Guilhem G., Bouillot J.L., Hugol D., Coussieu C., Basdevant A., Bar Hen A., Bedossa P. (2006). Increased infiltration of macrophages in omental adipose tissue is associated with marked hepatic lesions in morbid human obesity. Diabetes.

[54] Hardy O.T., Perugini R.A., Nicoloro S.M., Gallagher-Dorval K., Puri V., Straubhaar J., Czech M.P. (2011). Body mass index-independent inflammation in omental adipose tissue associated with insulin resistance in morbid obesity. Surg. Obes. Relat. Dis..

[55] van der Kolk B.W., Kalafati M., Adriaens M., van Greevenbroek M.M.J., Vogelzangs N., Saris W.H.M., Astrup A., Valsesia A., Langin D., van der Kallen C.J.H. (2019). Subcutaneous adipose tissue and systemic inflammation are associated with peripheral but not hepatic insulin resistance in humans. Diabetes.

[56] Kratz M., Coats B.R., Hisert K.B., Hagman D., Mutskov V., Peris E., Schoenfelt K.Q., Kuzma J.N., Larson I., Billing P.S. (2014). Metabolic dysfunction drives a mechanistically distinct proinflammatory phenotype in adipose tissue macrophages. Cell Metab..

[57] Xu X., Grijalva A., Skowronski A., van Eijk M., Serlie M.J., Ferrante A.W. (2013). Obesity activates a program of lysosomal-dependent lipid metabolism in adipose tissue macrophages independently of classic activation. Cell Metab..

[58] Russo L., Lumeng C.N. (2018). Properties and functions of adipose tissue macrophages in obesity. Immunology.

[59] Kolliniati O., Ieronymaki E., Vergadi E., Tsatsanis C. (2022). Metabolic regulation of macrophage activation. J. Innate Immun..

[60] Burska A.N., Sakthiswary R., Sattar N. (2015). Effects of tumour necrosis factor antagonists on insulin sensitivity/resistance in rheumatoid arthritis: A systematic review and meta-Analysis. PLoS ONE.

[61] Ofei F., Hurel S., Newkirk J., Sopwith M., Taylor R. (1996). Effects of an engineered human anti-TNF-alpha antibody (CDP571) on insulin sensitivity and glycemic control in patients with NIDDM. Diabetes.

[62] Wascher T.C., Lindeman J.H., Sourij H., Kooistra T., Pacini G., Roden M. (2011). Chronic TNF-α neutralization does not improve insulin resistance or endothelial function in “healthy” men with metabolic syndrome. Mol. Med..

[63] Larsen C.M., Faulenbach M., Vaag A., Vølund A., Ehses J.A., Seifert B., Mandrup-Poulsen T., Donath M.Y. (2007). Interleukin-1-receptor antagonist in type 2 diabetes mellitus. N. Engl. J. Med..

[64] Yuan M., Konstantopoulos N., Lee J., Hansen L., Li Z.W., Karin M., Shoelson S.E. (2001). Reversal of obesity- and diet-induced insulin resistance with salicylates or targeted disruption of Ikkbeta. Science.

[65] Zhang H., Desai N.N., Olivera A., Seki T., Brooker G., Spiegel S. (1991). Sphingosine-1-phosphate, a novel lipid, involved in cellular proliferation. J. Cell Biol..

[66] Fyrst H., Saba J.D. (2010). An update on sphingosine-1-phosphate and other sphingolipid mediators. Nat. Chem. Biol..

[67] Ancellin N., Colmont C., Su J., Li Q., Mittereder N., Chae S.S., Stefansson S., Liau G., Hla T. (2002). Extracellular export of sphingosine kinase-1 enzyme. Sphingosine 1-phosphate generation and the induction of angiogenic vascular maturation. J. Biol. Chem..

[68] Chaurasia B., Summers S.A. (2021). Ceramides in Metabolism: Key Lipotoxic Players. Annu. Rev. Physiol..

[69] Igarashi N., Okada T., Hayashi S., Fujita T., Jahangeer S., Nakamura S. (2003). Sphingosine kinase 2 is a nuclear protein and inhibits DNA synthesis. J. Biol. Chem..

[70] Yatomi Y., Igarashi Y., Yang L., Hisano N., Qi R., Asazuma N., Satoh K., Ozaki Y., Kume S. (1997). Sphingosine 1-phosphate, a bioactive sphingolipid abundantly stored in platelets, is a normal constituent of human plasma and serum. J. Biochem..

[71] Xu N., Dahlbäck B. (1999). A novel human apolipoprotein (apoM). J. Biol. Chem..

[72] Spiegel S., Milstien S. (2003). Sphingosine-1-phosphate: An enigmatic signalling lipid. Nat. Rev. Mol. Cell Biol..

[73] Ishii I., Fukushima N., Ye X., Chun J. (2004). Lysophospholipid receptors: Signaling and biology. Annu. Rev. Biochem..

[74] Bravo G., Cedeño R.R., Casadevall M.P., Ramió-Torrentà L. (2022). Sphingosine-1-phosphate (S1P) and S1P signaling pathway modulators, from current insights to future perspectives. Cells.

[75] Michaud J., Im D.S., Hla T. (2010). Inhibitory role of sphingosine 1-phosphate receptor 2 in macrophage recruitment during inflammation. J. Immunol..

[76] Hou L., Yang L., Chang N., Zhao X., Zhou X., Dong C., Liu F., Yang L., Li L. (2020). Macrophage sphingosine 1-phosphate receptor 2 blockade attenuates liver inflammation and fibrogenesis triggered by NLRP3 inflammasome. Front. Immunol..

[77] Awojoodu A.O., Ogle M.E., Sefcik L.S., Bowers D.T., Martin K., Brayman K.L., Lynch K.R., Peirce-Cottler S.M., Botchwey E. (2013). Sphingosine 1-phosphate receptor 3 regulates recruitment of anti-inflammatory monocytes to microvessels during implant arteriogenesis. Proc. Natl. Acad. Sci. USA.

[78] Murakami K., Kohno M., Kadoya M., Nagahara H., Fujii W., Seno T., Yamamoto A., Oda R., Fujiwara H., Kubo T. (2014). Knock out of S1P3 receptor signaling attenuates inflammation and fibrosis in bleomycin-induced lung injury mice model. PLoS ONE.

[79] Okamoto H., Takuwa N., Yokomizo T., Sugimoto N., Sakurada S., Shigematsu H., Takuwa Y. (2000). Inhibitory regulation of Rac activation, membrane ruffling, and cell migration by the G protein-coupled sphingosine-1-phosphate receptor EDG5 but not EDG1 or EDG3. Mol. Cell. Biol..

[80] Yamaguchi H., Kitayama J., Takuwa N., Arikawa K., Inoki I., Takehara K., Nagawa H., Takuwa Y. (2003). Sphingosine-1-phosphate receptor subtype-specific positive and negative regulation of Rac and haematogenous metastasis of melanoma cells. Biochem. J..

[81] Weigert A., Weis N., Brüne B. (2009). Regulation of macrophage function by sphingosine-1-phosphate. Immunobiology.

[82] Hait N.C., Allegood J., Maceyka M., Strub G.M., Harikumar K.B., Singh S.K., Luo C., Marmorstein R., Kordula T., Milstien S. (2009). Regulation of histone acetylation in the nucleus by sphingosine-1-phosphate. Science.

[83] Strub G.M., Paillard M., Liang J., Gomez L., Allegood J.C., Hait N.C., Maceyka M., Price M.M., Chen Q., Simpson D.C. (2011). Sphingosine-1-phosphate produced by sphingosine kinase 2 in mitochondria interacts with prohibitin 2 to regulate complex IV assembly and respiration. FASEB J..

[84] Kappos L., Radue E.W., O’Connor P., Polman C., Hohlfeld R., Calabresi P., Selmaj K., Agoropoulou C., Leyk M., Zhang-Auberson L. (2010). A placebo-controlled trial of oral fingolimod in relapsing multiple sclerosis. N. Engl. J. Med..

[85] Chiba K., Yanagawa Y., Masubuchi Y., Kataoka H., Kawaguchi T., Ohtsuki M., Hoshino Y. (1998). FTY720, a novel immunosuppressant, induces sequestration of circulating mature lymphocytes by acceleration of lymphocyte homing in rats. I. FTY720 selectively decreases the number of circulating mature lymphocytes by acceleration of lymphocyte homing. J. Immunol..

[86] Lahiri S., Park H., Laviad E.L., Lu X., Bittman R., Futerman A.H. (2009). Ceramide synthesis is modulated by the sphingosine analog FTY720 via a mixture of uncompetitive and noncompetitive inhibition in an Acyl-CoA chain length-dependent manner. J. Biol. Chem..

[87] Ogretmen B. (2018). Sphingolipid metabolism in cancer signalling and therapy. Nat. Rev. Cancer.

[88] Cao M., Ji C., Zhou Y., Huang W., Ni W., Tong X., Wei J.F. (2018). Sphingosine kinase inhibitors: A patent review. Int. J. Mol. Med..

[89] Bagdanoff J.T., Donoviel M.S., Nouraldeen A., Carlsen M., Jessop T.C., Tarver J., Aleem S., Dong L., Zhang H., Boteju L. (2010). Inhibition of sphingosine 1-phosphate lyase for the treatment of rheumatoid arthritis: Discovery of (E)-1-(4-((1R,2S,3R)-1,2,3,4-tetrahydroxybutyl)-1H-imidazol-2-yl)ethanone oxime (LX2931) and (1R,2S,3R)-1-(2-(isoxazol-3-yl)-1H-imidazol-4-yl)butane-1,2,3,4-tetraol (LX2932). J. Med. Chem..

[90] Vilas-Boas E.A., Almeida D.C., Roma L.P., Ortis F., Carpinelli A.R. (2021). Lipotoxicity and β-cell failure in type 2 diabetes: Oxidative stress lnked to NADPH oxidase and ER stress. Cells.

[91] Blüher M., Kahn B.B., Kahn C.R. (2003). Extended longevity in mice lacking the insulin receptor in adipose tissue. Science.

[92] Lauro D., Kido Y., Castle A.L., Zarnowski M.J., Hayashi H., Ebina Y., Accili D. (1998). Impaired glucose tolerance in mice with a targeted impairment of insulin action in muscle and adipose tissue. Nat. Genet..

[93] Michael M.D., Kulkarni R.N., Postic C., Previs S.F., Shulman G.I., Magnuson M.A., Kahn C.R. (2000). Loss of insulin signaling in hepatocytes leads to severe insulin resistance and progressive hepatic dysfunction. Mol. Cell.

[94] Guitton J., Bandet C.L., Mariko M.L., Tan-Chen S., Bourron O., Benomar Y., Hajduch E., Le Stunff H. (2020). Sphingosine-1-phosphate metabolism in the regulation of obesity/type 2 diabetes. Cells.

[95] Obinata H., Hla T. (2019). Sphingosine 1-phosphate and inflammation. Int Immunol.

[96] Wang J., Badeanlou L., Bielawski J., Ciaraldi T.P., Samad F. (2014). Sphingosine kinase 1 regulates adipose proinflammatory responses and insulin resistance. Am. J. Physiol. Endocrinol. Metab..

[97] Chaurasia B., Kaddai V.A., Lancaster G.I., Henstridge D.C., Sriram S., Galam D.L., Gopalan V., Prakash K.N., Velan S.S., Bulchand S. (2016). Adipocyte ceramides regulate subcutaneous adipose browning, inflammation, and metabolism. Cell Metab..

[98] Bruce C.R., Risis S., Babb J.R., Yang C., Lee-Young R.S., Henstridge D.C., Febbraio M.A. (2013). The sphingosine-1-phosphate analog FTY720 reduces muscle ceramide content and improves glucose tolerance in high fat-fed male mice. Endocrinology.

[99] Sakurai Y., Kubota N., Yamauchi T., Kadowaki T. (2021). Role of insulin resistance in MAFLD. Int. J. Mol. Sci..

[100] Brown M.S., Goldstein J.L. (2008). Selective versus total insulin resistance: A pathogenic paradox. Cell Metab..

[101] Wigger D., Schumacher F., Schneider-Schaulies S., Kleuser B. (2021). Sphingosine 1-phosphate metabolism and insulin signaling. Cell Signal..

[102] Monetti M., Levin M.C., Watt M.J., Sajan M.P., Marmor S., Hubbard B.K., Stevens R.D., Bain J.R., Newgard C.B., Farese R.V. (2007). Dissociation of hepatic steatosis and insulin resistance in mice overexpressing DGAT in the liver. Cell Metab..

[103] Summers S.A., Garza L.A., Zhou H., Birnbaum M.J. (1998). Regulation of insulin-stimulated glucose transporter GLUT4 translocation and Akt kinase activity by ceramide. Mol. Cell. Biol..

[104] Raichur S., Brunner B., Bielohuby M., Hansen G., Pfenninger A., Wang B., Bruning J.C., Larsen P.J., Tennagels N. (2019). The role of C16:0 ceramide in the development of obesity and type 2 diabetes: CerS6 inhibition as a novel therapeutic approach. Mol. Metab..

[105] Grammatikos G., Mühle C., Ferreiros N., Schroeter S., Bogdanou D., Schwalm S., Hintereder G., Kornhuber J., Zeuzem S., Sarrazin C. (2014). Serum acid sphingomyelinase is upregulated in chronic hepatitis C infection and non alcoholic fatty liver disease. Biochim. Biophys. Acta.

[106] Fayyaz S., Henkel J., Japtok L., Krämer S., Damm G., Seehofer D., Püschel G.P., Kleuser B. (2014). Involvement of sphingosine 1-phosphate in palmitate-induced insulin resistance of hepatocytes via the S1P2 receptor subtype. Diabetologia.

[107] Aji G., Huang Y., Ng M.L., Wang W., Lan T., Li M., Li Y., Chen Q., Li R., Yan S. (2020). Regulation of hepatic insulin signaling and glucose homeostasis by sphingosine kinase 2. Proc. Natl. Acad. Sci. USA.

[108] Ma M.M., Chen J.L., Wang G.G., Wang H., Lu Y., Li J.F., Yi J., Yuan Y.J., Zhang Q.W., Mi J. (2007). Sphingosine kinase 1 participates in insulin signalling and regulates glucose metabolism and homeostasis in KK/Ay diabetic mice. Diabetologia.

[109] Chakrabarty S., Bui Q., Badeanlou L., Hester K., Chun J., Ruf W., Ciaraldi T.P., Samad F. (2022). S1P/S1PR3 signalling axis protects against obesity-induced metabolic dysfunction. Adipocyte.

[110] Kurano M., Tsukamoto K., Shimizu T., Kassai H., Nakao K., Aiba A., Hara M., Yatomi Y. (2020). Protection against insulin resistance by apolipoprotein M/sphingosine-1-phosphate. Diabetes.

[111] Hashimoto T., Igarashi J., Kosaka H. (2009). Sphingosine kinase is induced in mouse 3T3-L1 cells and promotes adipogenesis. J. Lipid Res..

[112] Moon M.H., Jeong J.K., Lee Y.J., Seol J.W., Park S.Y. (2014). Sphingosine-1-phosphate inhibits the adipogenic differentiation of 3T3-L1 preadipocytes. Int. J. Mol. Med..

[113] Hashimoto Y., Matsuzaki E., Higashi K., Takahashi-Yanaga F., Takano A., Hirata M., Nishimura F. (2015). Sphingosine-1-phosphate inhibits differentiation of C3H10T1/2 cells into adipocyte. Mol. Cell. Biochem..

[114] Kendall M.R., Hupfeld C.J. (2008). FTY720, a sphingosine-1-phosphate receptor modulator, reverses high-fat diet-induced weight gain, insulin resistance and adipose tissue inflammation in C57BL/6 mice. Diabetes Obes. Metab..

[115] Moon M.H., Jeong J.K., Lee J.H., Park Y.G., Lee Y.J., Seol J.W., Park S.Y. (2012). Antiobesity activity of a sphingosine 1-phosphate analogue FTY720 observed in adipocytes and obese mouse model. Exp. Mol. Med..

[116] Ishii I., Ye X., Friedman B., Kawamura S., Contos J.J., Kingsbury M.A., Yang A.H., Zhang G., Brown J.H., Chun J. (2002). Marked perinatal lethality and cellular signaling deficits in mice null for the two sphingosine 1-phosphate (S1P) receptors, S1P(2)/LP(B2)/EDG-5 and S1P(3)/LP(B3)/EDG-3. J. Biol. Chem..

[117] Ishii I., Friedman B., Ye X., Kawamura S., McGiffert C., Contos J.J., Kingsbury M.A., Zhang G., Brown J.H., Chun J. (2001). Selective loss of sphingosine 1-phosphate signaling with no obvious phenotypic abnormality in mice lacking its G protein-coupled receptor, LP(B3)/EDG-3. J. Biol. Chem..

[118] Jeong J.K., Moon M.H., Park S.Y. (2015). Modulation of the expression of sphingosine 1-phosphate 2 receptors regulates the differentiation of pre-adipocytes. Mol. Med. Rep..

[119] Schmitz-Peiffer C., Craig D.L., Biden T.J. (1999). Ceramide generation is sufficient to account for the inhibition of the insulin-stimulated PKB pathway in C2C12 skeletal muscle cells pretreated with palmitate. J. Biol. Chem..

[120] Rapizzi E., Taddei M.L., Fiaschi T., Donati C., Bruni P., Chiarugi P. (2009). Sphingosine 1-phosphate increases glucose uptake through trans-activation of insulin receptor. Cell Mol. Life Sci..

[121] Pierucci F., Frati A., Battistini C., Matteini F., Iachini M.C., Vestri A., Penna F., Costelli P., Meacci E. (2018). Involvement of released sphingosine 1-phosphate/sphingosine 1-phosphate receptor axis in skeletal muscle atrophy. Biochim. Biophys. Acta Mol. Basis. Dis..

[122] Bruce C.R., Risis S., Babb J.R., Yang C., Kowalski G.M., Selathurai A., Lee-Young R.S., Weir J.M., Yoshioka K., Takuwa Y. (2012). Overexpression of sphingosine kinase 1 prevents ceramide accumulation and ameliorates muscle insulin resistance in high-fat diet-fed mice. Diabetes.

[123] Carey A.L., Steinberg G.R., Macaulay S.L., Thomas W.G., Holmes A.G., Ramm G., Prelovsek O., Hohnen-Behrens C., Watt M.J., James D.E. (2006). Interleukin-6 increases insulin-stimulated glucose disposal in humans and glucose uptake and fatty acid oxidation in vitro via AMP-activated protein kinase. Diabetes.

[124] Ross J.S., Hu W., Rosen B., Snider A.J., Obeid L.M., Cowart L.A. (2013). Sphingosine kinase 1 is regulated by peroxisome proliferator-activated receptor α in response to free fatty acids and is essential for skeletal muscle interleukin-6 production and signaling in diet-induced obesity. J. Biol. Chem..

[125] Leibiger I.B., Leibiger B., Berggren P.O. (2008). Insulin signaling in the pancreatic beta-cell. Annu. Rev. Nutr..

[126] Withers D.J., Burks D.J., Towery H.H., Altamuro S.L., Flint C.L., White M.F. (1999). Irs-2 coordinates Igf-1 receptor-mediated beta-cell development and peripheral insulin signalling. Nat. Genet..

[127] Maedler K., Spinas G.A., Dyntar D., Moritz W., Kaiser N., Donath M.Y. (2001). Distinct effects of saturated and monounsaturated fatty acids on beta-cell turnover and function. Diabetes.

[128] Hasan N.M., Longacre M.J., Stoker S.W., Kendrick M.A., Druckenbrod N.R., Laychock S.G., Mastrandrea L.D., MacDonald M.J. (2012). Sphingosine kinase 1 knockdown reduces insulin synthesis and secretion in a rat insulinoma cell line. Arch. Biochem. Biophys..

[129] Cantrell Stanford J., Morris A.J., Sunkara M., Popa G.J., Larson K.L., Özcan S. (2012). Sphingosine 1-phosphate (S1P) regulates glucose-stimulated insulin secretion in pancreatic beta cells. J. Biol. Chem..

[130] Zhao Z., Choi J., Zhao C., Ma Z.A. (2012). FTY720 normalizes hyperglycemia by stimulating β-cell in vivo regeneration in db/db mice through regulation of cyclin D3 and p57(KIP2). J. Biol. Chem..

[131] Imasawa T., Koike K., Ishii I., Chun J., Yatomi Y. (2010). Blockade of sphingosine 1-phosphate receptor 2 signaling attenuates streptozotocin-induced apoptosis of pancreatic beta-cells. Biochem. Biophys. Res. Commun..

[132] Kurano M., Tsuneyama K., Morimoto Y., Nishikawa M., Yatomi Y. (2019). Apolipoprotein M suppresses the phenotypes of IgA nephropathy in hyper-IgA mice. FASEB J..

[133] Hajny S., Christoffersen M., Dalila N., Nielsen L.B., Tybjærg-Hansen A., Christoffersen C. (2020). Apolipoprotein M and risk of type 2 diabetes. J. Clin. Endocrinol. Metab..

